# A predictive model for early clinical diagnosis of spinal tuberculosis based on conventional laboratory indices: A multicenter real-world study

**DOI:** 10.3389/fcimb.2023.1150632

**Published:** 2023-03-24

**Authors:** Xiaojiang Hu, Guang Zhang, Hongqi Zhang, Mingxing Tang, Shaohua Liu, Bo Tang, Dongcheng Xu, Chengran Zhang, Qile Gao

**Affiliations:** ^1^ Department of Spine Surgery and Orthopaedics, Xiangya Hospital, Central South University, Changsha, China; ^2^ National Clinical Research Center for Geriatric Disorders, Xiangya Hospital, Central South University, Changsha, China

**Keywords:** spinal tuberculosis, early diagnosis, nomogram, predictive model, conventional laboratory indices

## Abstract

**Background:**

Early diagnosis of spinal tuberculosis (STB) remains challenging. The aim of this study was to develop a predictive model for the early diagnosis of STB based on conventional laboratory indicators.

**Method:**

The clinical data of patients with suspected STB in four hospitals were included, and variables were screened by Lasso regression. Eighty-five percent of the cases in the dataset were randomly selected as the training set, and the other 15% were selected as the validation set. The diagnostic prediction model was established by logistic regression in the training set, and the nomogram was drawn. The diagnostic performance of the model was verified in the validation set.

**Result:**

A total of 206 patients were included in the study, including 105 patients with STB and 101 patients with NSTB. Twelve variables were screened by Lasso regression and modeled by logistic regression, and seven variables (TB.antibody, IGRAs, RBC, Mono%, RDW, AST, BUN) were finally included in the model. AUC of 0.9468 and 0.9188 in the training and validation cohort, respectively.

**Conclusion:**

In this study, we developed a prediction model for the early diagnosis of STB which consisted of seven routine laboratory indicators.

## 1 Introduction

Tuberculosis (TB) is a chronic infectious disease caused by infection with Mycobacterium tuberculosis, and it is the oldest infectious disease that has been identified in humans (Scorrano et al., 2022). According to the latest Global TB Report published by the World Health Organization in 2021, in 2020, an estimated 10 million people worldwide had tuberculosis. This included 5.6 million men, 3.3 million women, and 1.1 million children. In 2020, an estimated 1.5 million people died from TB, making it the second highest infectious disease killer after novel coronavirus pneumonia (World Health Organization, 2021).

Spinal tuberculosis (STB) is an extrapulmonary manifestation of tuberculosis that occurs as a secondary infection caused by Mycobacterium tuberculosis in the spinal vertebrae and adnexal tissues. STB accounts for approximately 2% of all tuberculosis cases and 50% of all osteoarticular tuberculosis cases. Worldwide, the annual incidence of spinal tuberculosis exceeds 100,000 (Khanna and Sabharwal, 2019a). STB can cause bone destruction, collapse, and fracture of the vertebral body, which can compress the spinal cord and cause paraplegia in approximately 10%-30% of affected patients (Hristea et al., 2008). The devastating and disabling effects of STB cannot be ignored, and early diagnosis of STB is essential to reduce the incidence of postoperative complications in patients. The gold standard for the diagnosis of spinal tuberculosis involves invasive tests such as surgical needle biopsy puncture to obtain a specimen of the lesion; diagnosis is then determined by using techniques such as Mycobacterium tuberculosis culture and Xpert, but this often occurs late in the patient’s hospitalization (Held et al., 2014; Beltran et al., 2022). If the patient receives an accurate diagnosis early in the course of the disease, the spine surgeon can recommend early treatment with anti-tuberculosis drugs, thereby improving the patient’s prognosis (Li et al., 2020).

However, early diagnosis in patients with spinal tuberculosis has been difficult, and cases are frequently missed or misdiagnosed (Mann et al., 2021). Patients with spinal tuberculosis typically lack early clinical symptoms; it is also difficult to distinguish STB from diseases such as septic spondylitis and spinal tumors, which have similar imaging manifestations as spinal tuberculosis, including bone destruction (Liu et al., 2021; Rule et al., 2021). Moreover, routine laboratory tests of inflammatory markers such as blood sedimentation, white blood cell count, and C-reactive protein are not specific for the diagnosis of tuberculosis, and these markers are also elevated in septic spondylitis (Liu et al., 2022). Interferon gamma release assays (IGRAs) have shown better diagnostic efficacy, as confirmed by our previously published study (Hu et al., 2022). However, we also observed that IGRAs alone do not meet the clinical need for diagnostic accuracy in spinal tuberculosis. Additionally, IGRAs cannot distinguish between active and latent tuberculosis and show no correlation with the duration of disease, limiting its use in the diagnosis of spinal tuberculosis (Hamada et al., 2021).

In our present study, we collected routine laboratory serology upon admission of patients with spinal tuberculosis, screened variables by lasso regression, built a model by logistic regression, and drew a nomogram for the early diagnosis of spinal tuberculosis based on a model used for patients in a multicenter retrospective study.

## 2 Method

### 2.1 Inclusion and exclusion criteria, and diagnosis criteria

This study included 206 patients with suspected spinal tuberculosis who were admitted to the Xiangya Hospital of Central South University, Xiangya Boai Hospital, Changsha First Hospital, and Hunan Chest Hospital from January 2016 to October 2022. The data of the included patients were taken from the first routine serological examination at the time of patient admission for treatment. The inclusion criteria were as follows: 1. CT and MRI reports of patients included in the study showed that patients had suspected spinal tuberculosis, including irregular lytic lesions or bone destruction that affect the vertebral body, endplate, or adjacent margin of the intervertebral disc, featuring fragmentary destruction, osteolytic destruction, and local destruction with sclerotic margins. Intervertebral disc destruction or decreased disc height, calcification or bone fragment formation accompanied by abscesses, vertebral collapse or posterior convex deformity, as well as soft tissue involvement and large paravertebral abscesses (Khanna and Sabharwal, 2019b); 2. Patients had complete clinical examination data. The exclusion criteria were as follows: 1. concomitant autoimmune diseases or HIV; 2. infections in multiple sites; and 3. patients with severe systemic chronic underlying diseases.

Patients included in this study were diagnosed with spinal tuberculosis if they met one of the following criteria (Pai et al., 2016): 1. positive culture for Mycobacterium tuberculosis from surgical needle biopsy; 2. positive molecular tests for Mycobacterium tuberculosis (including Xpert and mNGS); 3. relevant pathologic histologic features (at least one of the following features: caseous necrosis, positive acid-fast staining, and granulomatous inflammation) Meanwhile, anti-tuberculosis treatment of patients is effective.

Patients were classified into the NSTB group based on the following criteria: 1. microbiological evidence that the infection was caused by other bacteria, fungi or viruses; 2. pathological histological diagnosis of other lesions of the spine (including septicemia and tumors).

Ethical Approval: Institutional Review Board approval was obtained (IRB#: 201303232). This study was approved by the Ethics Committee of Xiangya Hospital Central South University, and written informed consent was obtained from all patients.

### 2.2 Routine laboratory test data collection

We collected the following routine laboratory test data at the time of admission for the study population: tubercle bacillus antibody (TB.antibody), interferon-γ release assays (IGRAs), white blood cell (WBC), red blood cell (RBC), hemoglobin (HGB), platelet (PLT), neutrophil (Neut), lymphocyte (lymph), eosinophil (EO), basophil (BASO), monocyte (Mono), neutrophil % (Neut%), lymphocyte % (Lymph%), basophil % (BASO%), eosinophil% (Eo%), monocyte % (Mono%), red blood cell distribution width (RDW), platelet volume (PCT), mean platelet volume (MPV), total protein (TP), albumin (A), globulin (G), albumin/globulin (AG), alanine aminotransferase (ALT), aspartate aminotransferase (AST), blood urea nitrogen (BUN), creatinine (Cr), triglyceride (TG), cholesterol (Chol), high-density lipoprotein (HDL), low-density lipoprotein (LDL), glucose (BS), prothrombin time (PT), activated partial thromboplatin time (APTT), international normalized ratio (INR), D dimer (DD), erythrocyte sedimentation rate (ESR), C-reactive protein (CRP).

### 2.3 Statistical analysis

All statistical analyses and graphing were based on R version 4.2.2. The “CBCgrps” (Zhang et al., 2017) package was used to draw baseline tables in which categorical variables were analyzed for differences in distribution between the two groups using chi-square tests (or Fisher’s exact probability method). Continuous data that conformed to a normal distribution are described as the mean (± standard deviation) and analyzed for differences between two groups using t tests, and data that did not conform to a normal distribution are described as the median (percentile), and differences between the two groups were analyzed using rank sum tests. The data that did not conform to the normal distribution are described as the median (percentile), and the differences between the two groups were analyzed using the rank sum test. The results of the comparison between the groups are expressed using p values. Heatmaps of correlations between variables were calculated and plotted using the “corrplot” package. The selection of variables was based on Lasso regression, and the continuous variables entered into the Lasso regression were standardized using the min-max transformation method. Lasso regression was performed by the “glmnet” package (nfolds were set to 20). The number of variables within one standard error was selected as the optimal number of variables. Eighty-five percent of the cases were randomly selected as the training set, and the rest of the cases were included in the validation set for the internal validation of the model. The “Rms” package was used to build the logistic regression model. The variables included in the logistic regression were selected based on the Lasso regression screening results, and variables lacking significance in the logistic model were excluded to refine the model. The calibration curves of the prediction model were plotted by the “caret” package. The column line plots were plotted using the “regplot” package, and the ROC curves were calculated and plotted using the “pROC” and “ggplot2” packages.

## 3 Result

### 3.1 Characteristics of the patient

A total of 206 patients with suspected spinal tuberculosis were enrolled according to the inclusion and exclusion criteria established for the study. We diagnosed 105 cases as spinal tuberculosis and 101 cases as non-STB according to the diagnostic criteria. The characteristics of the patients, routine laboratory data, and the results of statistical comparisons of these indicators between the STB and NSTB groups are presented in **Table 1**. There were no significant differences between the two groups in terms of age and sex. Detailed information on the underlying diseases included in the patients’ medical history is provided in **Table 2**. The NSTB group comprised 45 cases of pyogenic spinal infection, 20 cases of spinal brucellosis, 12 cases of spinal tumors, 18 cases of other non-specific infections, 4 cases of spinal fungal infections, and 2 cases of spinal viral infections (**Supplementary Figure 1**).

**Table 1:** Univariate analysis of patients and variables for included cases.

Variables	Total (n = 206)	NSTB (n = 101)	STB (n = 105)	p value
sex, n (%)				0.92
female	88 (43)	44 (44)	44 (42)	
male	118 (57)	57 (56)	61 (58)	
age, years	54.5 (46, 63)	56 (49, 63)	53 (44, 63)	0.47
TB.antibody, n (%)				< 0.001
negative	176 (85)	98 (97)	78 (74)	
positive	30 (15)	3 (3)	27 (26)	
IGRAs, n (%)				< 0.001
negative	77 (37)	72 (71)	5 (5)	
positive	129 (63)	29 (29)	100 (95)	
WBC, 10^9/L	6 (4.7, 7.68)	6.2 (4.8, 7.8)	5.8 (4.3, 7.5)	0.068
RBC, 10^12/L	4.11 ± 0.58	3.93 ± 0.55	4.27 ± 0.56	< 0.001
HGB, g/L	120.19 ± 16.12	117.94 ± 16.16	122.36 ± 15.85	0.049
PLT, 10^9/L	235 (184.25, 296.5)	231 (180, 293)	237 (189, 305)	0.721
Neut, 10^9/L	3.7 (2.8, 5.2)	3.8 (2.7, 5.1)	3.6 (2.9, 5.4)	0.682
lymph, 10^9/L	1.3 (1, 1.8)	1.5 (1.2, 2)	1.2 (0.9, 1.6)	< 0.001
EO, 10^9/L	0.1 (0.1, 0.2)	0.1 (0.1, 0.2)	0.1 (0.1, 0.2)	0.912
BASO, 10^9/L	0 (0, 0.03)	0 (0, 0.03)	0 (0, 0.03)	0.106
Mono, 10^9/L	0.5 (0.4, 0.7)	0.5 (0.4, 0.6)	0.6 (0.4, 0.7)	0.109
Neut%, %	65.75 (57.73, 71.6)	65.7 (55.7, 70.8)	65.8 (60.5, 72.1)	0.205
Lymph%, %	21.8 (16.95, 28)	22.9 (18.3, 31.3)	20.8 (15.9, 26.3)	0.012
BASO%, %	0.5 (0.4, 0.7)	0.5 (0.3, 0.6)	0.5 (0.4, 0.7)	0.233
Eo%, %	1.9 (1.1, 3.2)	1.8 (1.1, 3.1)	2 (1.1, 3.4)	0.519
Mono%, %	8.95 (7.23, 10.4)	8.4 (6.7, 9.6)	9.4 (8.1, 11.2)	< 0.001
RDW, %	14.05 (13.1, 14.8)	13.8 (13.2, 14.8)	14.1 (13.1, 14.8)	0.548
PCT, 10^9/L	0.2 (0.17, 0.25)	0.19 (0.17, 0.25)	0.2 (0.17, 0.25)	0.324
MPV, fL	8.46 (7.67, 9.26)	8.35 (7.65, 9.23)	8.69 (7.78, 9.32)	0.347
TP, g/L	70.1 (65.32, 73.7)	70.1 (65.1, 73.7)	70.1 (65.8, 73.7)	0.738
A, g/L	36.8 ± 4.44	36.03 ± 4.98	37.54 ± 3.72	0.015
G, g/L	32.45 (29, 37.27)	33.3 (28.9, 38.3)	31.6 (29.1, 35.8)	0.236
AG	1.1 (0.92, 1.3)	1.1 (0.9, 1.3)	1.2 (1, 1.4)	0.036
ALT, U/L	17.7 (11.9, 27.02)	19.3 (13.4, 33.4)	16.6 (11.1, 23.7)	0.041
AST, U/L	21.85 (16.8, 28.45)	23.1 (16.8, 28.7)	21 (16.8, 26.6)	0.359
BUN, mmol/L	4.68 (3.7, 6.04)	5.15 (3.81, 6.89)	4.59 (3.53, 5.58)	0.024
Cr, umol/L	71.55 (62, 84)	71.9 (62, 86.6)	71.1 (62, 81.2)	0.251
TG, mmol/L	1.23 (0.92, 1.65)	1.3 (0.95, 1.72)	1.17 (0.88, 1.5)	0.045
Chol, mmol/L	4.44 (3.63, 5.07)	4.58 (3.68, 5.14)	4.3 (3.61, 4.9)	0.294
HDL, mmol/L	1.02 (0.84, 1.21)	1.02 (0.84, 1.23)	1.04 (0.87, 1.15)	0.876
LDL, mmol/L	2.74 (2.28, 3.19)	2.79 (2.26, 3.31)	2.69 (2.3, 3.18)	0.447
BS, mmol/L	5.16 (4.74, 5.68)	5.27 (4.95, 5.89)	5.04 (4.71, 5.49)	0.002
PT, s	12.6 (11.72, 13.6)	12.4 (11.5, 13.4)	12.8 (12, 13.7)	0.052
APTT, s	31.35 (28.15, 35.4)	30.2 (27.3, 35)	32 (29.3, 35.5)	0.105
INR	1.03 (0.95, 1.09)	1.02 (0.94, 1.07)	1.03 (0.96, 1.09)	0.312
D_dimer, mg/L	0.27 (0.16, 0.46)	0.28 (0.18, 0.51)	0.26 (0.15, 0.42)	0.213
ESR, mm/h	70 (39, 101.75)	75 (41, 104)	66 (38, 94)	0.256
CRP, mg/L	15.58 (6, 41.47)	15.9 (5.71, 42.4)	12.6 (6.28, 37.8)	0.782

Continuous variables are expressed as mean ± standard deviation or median (25th percentile, 75th percentile).

**Table 2:** The clinical characteristics of recruited patients.

	STB (n = 105)	NSTB (n = 101)	P value
Diabetes, n (%)	9 (9)	14 (14)	0.325
Hypohepatia, n (%)	4 (4)	7 (7)	0.493
Renal Insufficiency, n (%)	1 (1)	6 (6)	0.061
Cancer, n (%)	2 (2)	12 (12)	0.01
pulmonary tuberculosis, n (%)	30 (29)	0 (0)	< 0.001

### 3.2 The performance of individual indicators in conventional laboratory tests for discriminating between STB and NSTB

**Figure 1:**
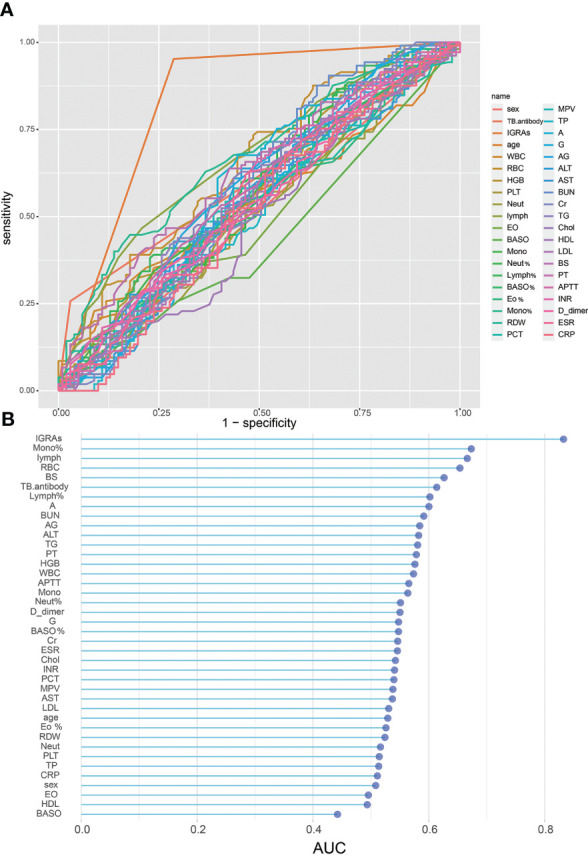
Single variable on STB diagnostic capability: **(A)**, ROC curve of single variable on STB diagnosis; **(B)**, AUC of single variables for STB diagnosis.

The results of the univariate analyses for STB patients and NSTB patients are presented in **Table 1**. We observed significant differences between the two groups in TB.antibody, RBC, mono%, lymph, and IGRAs (p < 0.001). In addition, the BS, Lymph%, A, BUN, AG, ALT, TG, and HGB were significantly different between the two groups (p < 0.05). We plotted ROC curves by using these univariate indicators (**Figure 1A**) and presented the results of their AUCs in a Lollipop Chart (**Figure 1B**). In the univariate analysis, we observed that only IGRAs had a high diagnostic validity with an AUC over 0.80, while the other indicators had a low univariate diagnostic validity, none of which exceeded 0.7.

### 3.3 Screening of variables by Lasso regression

**Figure 2:**
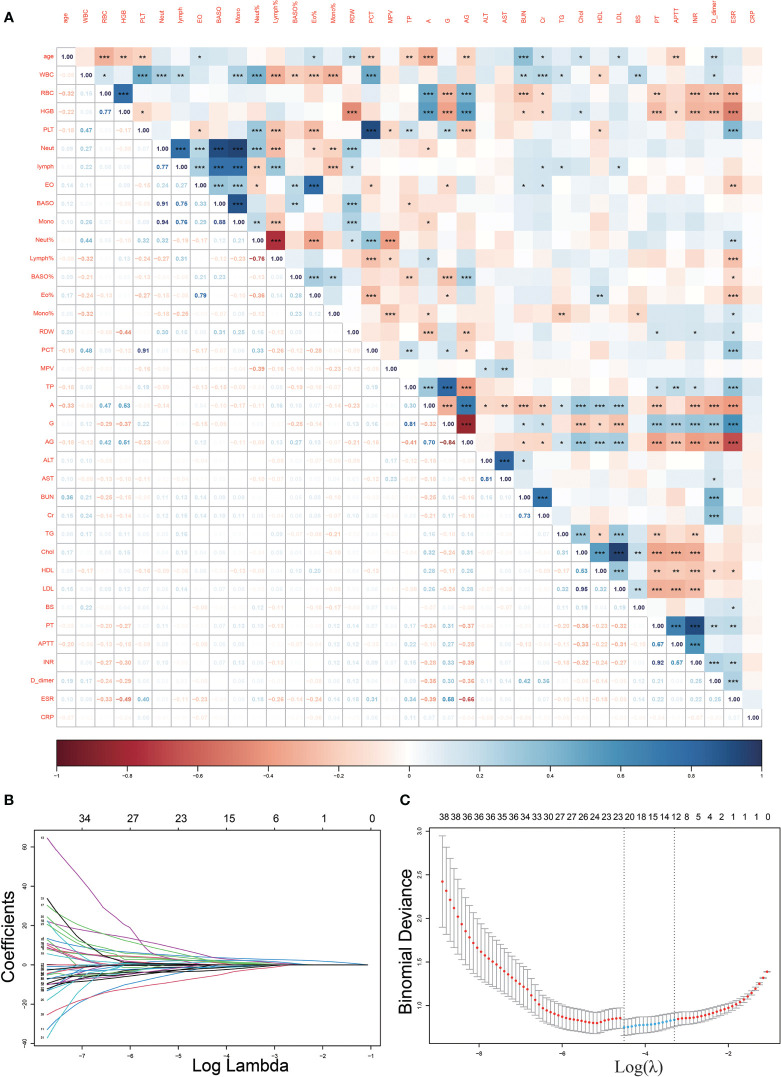
Heat map of correlations between variables and Lasso regression screening variables. **(A)**, The heat map of correlations between continuous variables demonstrates the existence of covariance between many variables (*p<0.05, **p<0.01; ***p<0.001); **(B, C)**, Lasso regression filtered out 12 variables from the 40.

We observed a strong correlation between some of the variables in our dataset (**Figure 2A**). To minimize the effect of covariance between weighted variables for each indicator in the linear model, we used Lasso regression to screen 40 variables (**Figures 2B, C**), and we identified12 indicators that could be used for subsequent predictive model fitting, including TB.antibody, IGRAs, WBC, RBC, Lymph%, Mono%, RDW, A, AST, BUN, TG, and PT.

### 3.4 Establishment of a predictive model for early diagnosis of STB and nomogram

**Figure 3:**
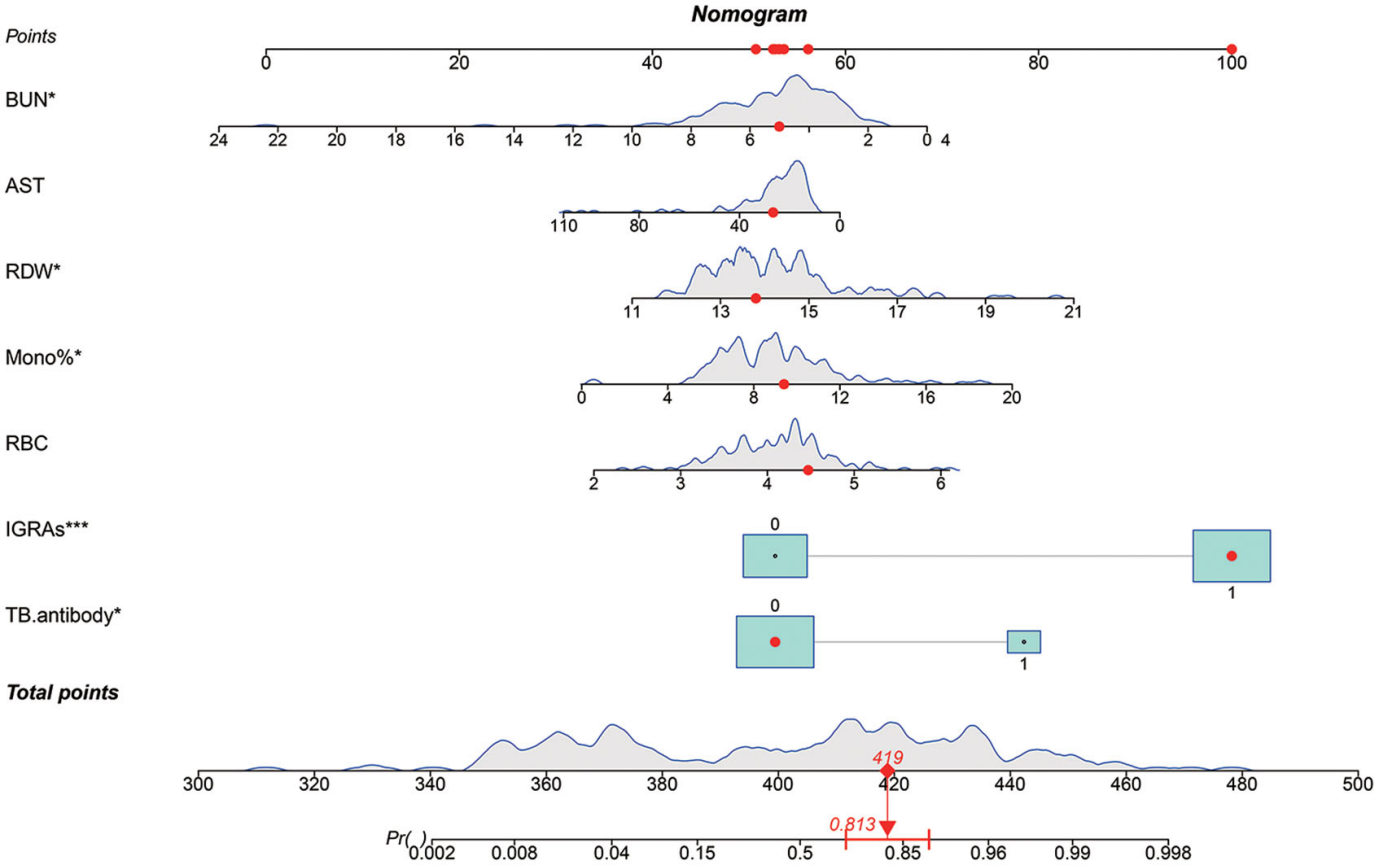
A predictive nomogram model for the early diagnosis of spinal tuberculosis. The red dots in the figure demonstrate the usage of the nomogram. A patient’s information was projected on the nomogram, and this patient had a final score of 419, corresponding to a probability of 0.813 (>0.5), so we judged this patient to have a final diagnosis of spinal tuberculosis (STB). The patient’s final diagnosis was consistent with our prediction.

We attempted to build logistic diagnostic models in the training set using twelve variables (TB.antibody, IGRAs, WBC, RBC, Lymph%, Mono%, RDW, A, AST, BUN, TG, PT) screened by Lasso regression, and we excluded variables that were not significant in the logistic regression model. We finally included seven variables, including TB.antibody, IGRAs, RBC, Mono%, RDW, AST, and BUN. We rebuilt the logistic regression model with these seven variables and drew a nomogram based on the logistic regression model that could be used in clinical practice (**Figure 3**).

### 3.5 Evaluation of a predictive model for early diagnosis of STB

**Figure 4:**
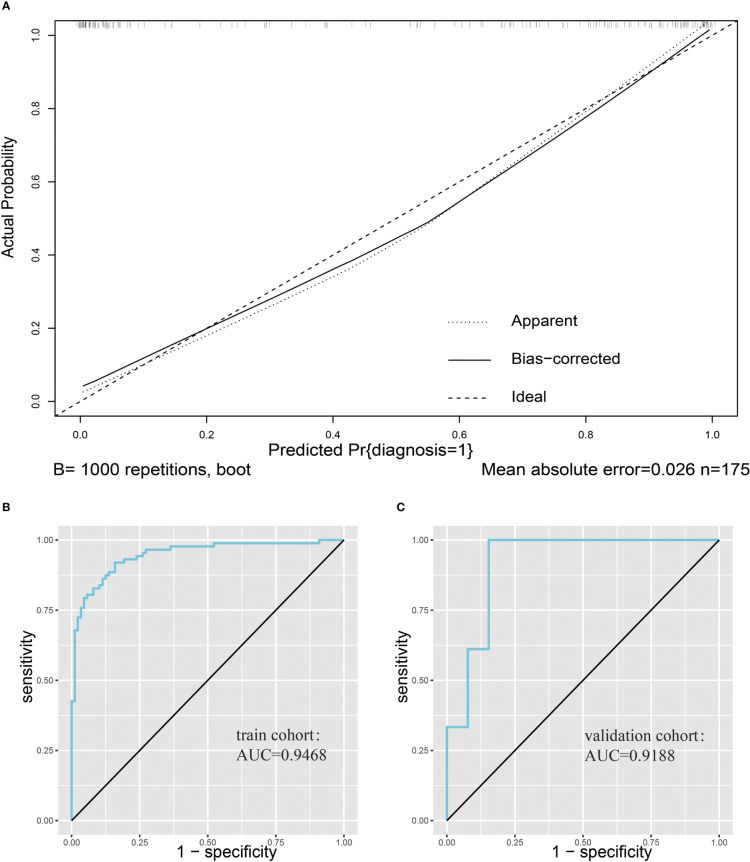
Evaluation of nomogram clinical prediction models. **(A)**, Calibration curve of the model. **(B)**, ROC curve of the model for the training cohort, and its AUC was 0.9468. **(C)**, ROC curve of the model for the validation cohort, and its AUC was 0.9188.

We first plotted the calibration curve of the model in the training set (**Figure 4A**). We observed the model has a good fit based on the calibration curve. In addition, we plotted the ROC curve of the model for the training cohort, and its AUC was 0.9468 (**Figure 4B**). Since the model was fitted based on the training cohort, we used an additional validation set to further evaluate the predictive performance of the model. After plotting the ROC curve of the prediction model in the validation cohort, we found that the AUC of the prediction model was 0.9188 (**Figure 4C**).

## 4 Discussion

Early diagnosis of spinal tuberculosis plays an important role in reducing complications and speeding up the recovery of patients (Chen et al., 2016). Early diagnosis of STB can guide physicians to use anti-tuberculosis drugs more effectively. Currently, the early diagnosis of STB before surgery relies on imaging and IGRAs. It is difficult to distinguish STB from septic spinal infections and some spinal tumors, which also manifest as bone destruction (Liu et al., 2021). IGRAs have been shown to be highly sensitive for the diagnosis of spinal tuberculosis (Hu et al., 2022) However, IGRAs have a low specificity for the diagnosis of STB due to their inability to differentiate between active and latent TB. Spine surgeons lack a tool that can be used for early differential diagnosis of spinal tuberculosis, leading to missed and misdiagnosed cases of spinal tuberculosis. Underdiagnosis of spinal tuberculosis delays the initiation of antituberculosis treatment in patients with spinal tuberculosis, preventing the early initiation and regular administration of antituberculosis treatment, whereas misdiagnosis of spinal tuberculosis results in patients with NSTB taking large amounts of antituberculosis drugs that are unnecessary (Dunn and Ben Husien, 2018). Antituberculosis drugs have considerable adverse effects, including hepatotoxicity, allergic reactions, peripheral neuritis, optic nerve damage, and hearing loss (Xu et al., 2017). In addition, the misuse of antituberculosis treatment may lead to the emergence of drug-resistant TB, which has been defined by the World Health Organization as a public health crisis; the incidence of drug-resistant TB has continued to increase in recent years, making the prevention of drug-resistant TB an urgent priority (World Health Organization, ).

In this study, we enrolled patients with suspected spinal tuberculosis from three hospitals. After removing data containing missing values and excluding patients that did not meet the inclusion and diagnostic criteria, a total of 206 eligible patients with suspected spinal tuberculosis were included for analysis. The final diagnosis was determined by postoperative pathogenic microbiology or pathology, and a total of 105 STB cases and 101 NSTB cases were identified. There were no significant differences in age or sex between patients in the STB and NSTB groups. Further univariate analysis of the results of 38 routine laboratory tests in STB patients and NSTB patients demonstrated that there were statistically significant differences between the two groups in TB.antibody, IGRAs, RBC, mono%, lymph, Lymph%, A, AG, BUN, BS, ALT, TG, and HGB (p < 0.05). Nevertheless, by plotting the ROC curves and calculating the AUC, we found that, except for IGRAs, which had good performance in the diagnosis of STB (AUC = 0.8326), other variables did not play a significant role in the diagnosis of STB (AUC < 0.7). An increasing number of studies have shown that one indicator alone is often unable to lead to a valid and accurate diagnosis, and the use of multiple indicators is more widely applicable. To meet practical clinical needs and minimize test costs, combining multiple routine laboratory tests to improve the early diagnostic efficacy of spinal tuberculosis can be applied for patients with spinal tuberculosis in underserved and remote areas.

Thirty-eight routine laboratory test variables and 2 other variables, including the age and sex of the patients, were included in this study. The variables selected are generally available at most hospitals to ensure practical application of the predictive model. Correlation analysis identified a high correlation between many of the variables, and this could be observed on the correlation heatmap. The problem of multicollinearity among the characteristics could not be resolved in the stepwise regression local optimum estimation of logistic regression (Bayman and Dexter, 2021). Therefore, we used Lasso regression, a regularization method widely used for model improvement and variable selection, for variable selection to optimize our model (Ternès et al., 2016). When multicollinearity existed in the original variables, Lasso regression could effectively screen the variables with multicollinearity (Fernández-Delgado et al., 2019). Using Lasso regression, we identified 12 variables that could be used for inclusion in the subsequent model.

Logistic regression was used to construct a predictive model for spinal tuberculosis. Twelve variables identified by Lasso regression were included in the logistic regression equation, and variables that were not significant in the logistic regression model were eliminated. Finally, seven variables were retained, including TB.antibody, IGRAs, RBC, Mono%, RDW, AST, and BUN were retained, and the model was refitted with these variables. Column line plots were drawn for better applicability to clinical practice. To evaluate the predictive model, we plotted calibration curves and ROC curves in a validation cohort different from the training cohort. The calibration curves showed a good fit, and the prediction model achieved an AUC value of 0.9188 in the validation cohort. The final model had good diagnostic validity. Based on our findings, increased levels of RBC, RDW, and mono% in patients could indicate a greater likelihood of STB diagnosis, whereas elevated BUN and AST levels may suggest a higher probability of NSTB diagnosis. In previously published studies, elevated RDW and mono% were commonly observed in patients with TB (Baynes et al., 1986). Patients with chronic TB infection often suffer from malnutrition, resulting in the development of anemia (Cobelens and Kerkhoff, 2021). However, in our study, the differential diagnosis of STB was often made in patients with other septic spinal infections and spinal tumors, who also had a poor nutritional status. This resulted in elevated levels of BUN and AST in both diseases, making it difficult to distinguish between them. Further studies are needed to better understand the distinction between these diseases.

In published studies, we noted that Liyi Chen et al. (2022) also attempted early diagnosis of spinal tuberculosis by drawing columnar maps, but they included fewer variables and lacked specific indicators for the diagnosis of spinal tuberculosis (IGRAs, TB antibody), and they did not develop strict inclusion and diagnostic criteria. A prospective study published in 2022 also diagnosed STB by combining multiple indicators, but we observed that this study included diagnostic indicators such as Xpert, acid-fast staining, and other indicators that occur late in the patient’s hospitalization (Qi et al., 2022); therefore, this study did not focus on the early diagnosis of STB. In addition, a recent study focused on a deep learning model for early diagnosis of spinal tuberculosis using imaging data analysis to determine the early diagnosis of spinal tuberculosis (Li et al., 2022), whereas our study focused on routine laboratory findings. In a follow-up study, we plan to combine both imaging data and routine laboratory findings to further optimize the early diagnosis prediction model of spinal tuberculosis through imaging histology and machine learning.

To enhance the generalizability of our model, we incorporated two distinct methods for IGRAs, namely QuantiFERON-TB Gold In-Tube and T-SPOT(^®^).TB, both of which share a similar underlying principle. Moreover, previous studies have highlighted the potential utility of the TBAg/PHA ratio as a means of improving diagnostic accuracy in tuberculosis (Wang et al., 2016; Luo et al., 2020), with particular promise demonstrated in the diagnosis of extrapulmonary disease (Wang et al., 2019). Although we were unable to include this variable in the current analysis, we eagerly anticipate the opportunity to assess the diagnostic efficacy of the TBAg/PHA ratio in T-SPOT(^®^).TB for spinal tuberculosis in a larger cohort of patients.

This study was a retrospective study, and due to missing data, we excluded some cases and other factors, such as calcitoninogen and body mass index, that might have been meaningful. We intend to conduct further prospective studies informed by our present results which will include complete clinical data and patient imaging data to further improve the early diagnosis of spinal tuberculosis.

## 5 Conclusion

In this study, we developed a nomogram to predict the occurrence of spinal tuberculosis for early diagnosis. Our model contained seven conventional laboratory indicators, including TB.antibody, IGRAs, RBC, Mono%, RDW, AST, and BUN. We hope to use this model to improve the early diagnosis of STB, especially in hospitals in poor and remote areas where testing is limited.

## Data availability statement

The raw data supporting the conclusions of this article will be made available by the authors, without undue reservation.

## Ethics statement

The studies involving human participants were reviewed and approved by the Ethics Committee of Xiangya Hospital Central South University. The patients/participants provided their written informed consent to participate in this study.

## Author contributions

XH and QG designed research, performed research, analyzed data, and wrote the paper. HZ, MT and SL developed the idea for the study. GZ, BT and DX collected the data. All authors contributed to the article and approved the submitted version.
