# Supplementary material for: A predictive model for early clinical diagnosis of spinal tuberculosis based on conventional laboratory indices: A multicenter real-world study
Source: Front Cell Infect Microbiol. 2023 Mar 24;13:1150632. doi: 10.3389/fcimb.2023.1150632 (PMC10080113; doi:10.3389/fcimb.2023.1150632)
Supplement: Supplementary file 1 [file DataSheet_1.pdf]

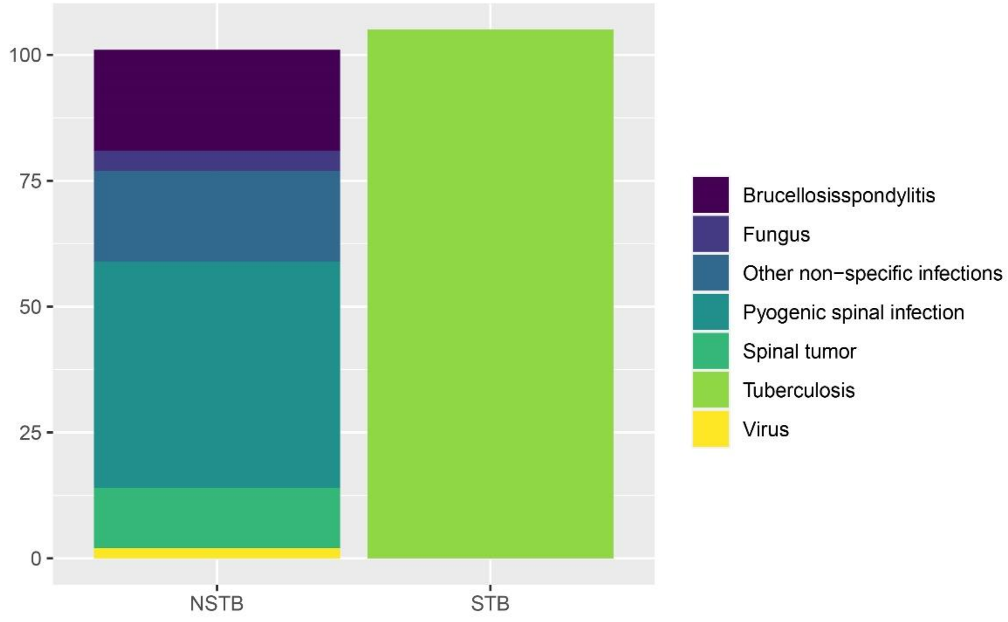

Supplementary Figure 1: The NSTB group comprised 45 cases of pyogenic spinal infection, 20 cases of spinal brucellosis, 12 cases of spinal tumors, 18 cases of other non-specific infections, 4 cases of spinal fungal infections, and 2 cases of spinal viral infections
